# Qualitative stress perfusion American Heart Association plot and outcome prediction using artificial intelligence

**DOI:** 10.1016/j.imu.2024.101537

**Published:** 2024

**Authors:** Ebraham Alskaf, Cian M. Scannell, Richard Crawley, Avan Suinesiaputra, PierGiorgio Masci, Alistair Young, Divaka Perera, Amedeo Chiribiri

**Affiliations:** a School of Biomedical Engineering & Imaging Sciences, King’s College London, United Kingdom; b Eindhoven University of Technology, Eindhoven, Netherlands

**Keywords:** American heart Association plot, All-cause mortality, Artificial intelligence, Image classification, Stress perfusion cardiac magnetic resonance

## 1 Introduction

Cardiac imaging is central to the diagnosis and risk stratification of coronary artery disease (CAD) beyond symptoms and clinical risk factors, by providing objective evidence of myocardial ischaemia and characterisation of coronary artery plaque. Literature has demonstrated the strong predictive power of non-invasive imaging modalities for CAD. Several studies have indicated that imaging adds significant prognostic value in predicting outcomes for patients with known or suspected CAD. This includes imaging focused on function and ischaemia assessment with cardiac magnetic resonance (CMR) [1], dobutamine stress echo-cardiography (DSE) [2], myocardial perfusion scanning (MPS) [3], or coronary anatomy imaging with coronary computed tomography angiography (CCTA) [4].

Stress perfusion CMR is a guideline-backed non-invasive imaging test for CAD assessment. The interpretation and clinical evaluation of CMR findings rely on visual assessment by expert readers. However, reported findings may exhibit a high level of inter-observer variability due to factors such as the level of training, the extent of ischaemia, and image quality, which could impact diagnostic accuracy [5].

On a separate note, the revolution of artificial intelligence (AI) and neural network development has entered the medical domain over the last decade. Emerging studies indicate that AI can detect traditionally challenging-to-identify or diagnose conditions and empower image classification. It has various applications in treatment, safety, patient adherence, administration, predictive analytics to explore patient datasets, predict the likelihood of certain diseases or outcomes, and precision medicine [6].

## 2 Literature review

There have been several studies examining the use of AI-based image classification techniques for stress perfusion cardiac imaging. One example used a fully automated deep learning approach to detect myocardial ischaemia from stress/rest myocardial perfusion single-photon emission computed tomography (SPECT) images, and it showed promising results in accurately identifying regions of ischaemia compared to manual interpretation by experts [7]. Another example used deep learning techniques to convert stress perfusion CMR images into coronary artery territory maps, which can aid in diagnosing CAD [8].

Earlier studies relied on popular basic machine learning approaches to classify stress perfusion imaging, such as multi-layer perceptron (MLP) and support vector machine (SVM), and MPS was the most popular imaging modality used. However, more recent studies showed the superior performance of convolutional neural network (CNN) achieving superior performance in stress perfusion image classification, and the application of AI-based classification extended to other imaging modalities, such as CMR [9].

The qualitative assessment of stress perfusion CMR images based on AHA segmentation still relies on expert readers and has not been automated. One important solution relies on stress perfusion quantification [10], which is aimed to standardise the assessment of ischaemia using CMR and make it more objective and less prone to observers’ bias, however, this promising technology is still on the horizon and the current clinical practice still relies primarily on qualitative assessment of stress perfusion images.

We aim in this study to use AI approach to: 1) automate the AHA-based qualitative stress perfusion plot and using CMR expert readers as a gold standard. 2) predict outcome using image data and clinical health records.

## 3 Methodology

### 3.1 Study design and population

This retrospective observational study focused on a large cohort of patients. Ethical approval for the study was obtained from the Research Ethics Committee under reference number 20/ES/0005, and the research adhered to the principles of the Declaration of Helsinki. Patients who underwent stress perfusion CMR at a single centre (St Thomas’ Hospital, King’s College London) were screened between April 2011 and March 2021. Only completed studies with comprehensive reports and available images were included.

The total number of all-cause mortality events was recorded for the entire population. The end of follow-up period was considered equivalent to the data collection date, which was the August 20, 2021, when all events were counted.

### 3.2 Inclusion and exclusion criteria

We included only patients with a complete adenosine stress perfusion study, good quality images, and comprehensive reports. Exclusion criteria comprised reports that were blinded for research purposes, discrepancies between main body text and summary findings, stress studies terminated due to complications, contraindications to stress agent use, mass perfusion studies, dobutamine stress studies, lung perfusion studies, poor responses to stress agents, and mis-labelled reports originally highlighted as perfusion studies but later found to be otherwise.

### 3.3 Data extraction

#### 3.3.1 Clinical data

Data extraction was primarily conducted using CogStack [11], a healthcare application framework that facilitates information extraction from unstructured data sources, such as electronic health records (EHRs). CogStack can process information locked in various unstructured formats (e.g., Word docs, PDFs, images, text fields) through natural language processing (NLP). This enables the retrieval of specific data by searching clinical text for terms using simple or complex syntax.

In this study, Cogstack was employed to extract baseline characteristics, including age and gender, as well as all-cause mortality using standard Elasticsearch query from structured dataset. Clinical risk factors were extracted using trained NLP models, as detailed in a prior publication [12].

Clinical variables encompassed age, gender, chronic kidney disease (CKD), hypertension (HTN), heart failure, smoking history, dyslipidaemia, diabetes mellitus (DM) and cerebrovascular accident (CVA). Age was expressed as a continuous variable, while other clinical variables were considered categorical and presented as binary variables.

#### 3.3.2 Image data

For image data extraction, stress perfusion CMR images comprised three series of frames representing three levels of slices: basal, mid and apical left ventricular (LV) slices. Legacy stress perfusion data included a mix of sequences, such as single bolus dual sequence with arterial input function (AIF), dual bolus single sequence, and single bolus single sequence. Two different vasodilators were used: Adenosine as a continuous infusion with the dose titrated to both patient heart rate and response (140/175/210 μg/kg/min), with imaging acquired at 3–6 min after commencement of the infusion, and Regadenoson as a 400 μg bolus with 10 ml saline flush, with imaging conducted at 2 min after administration.

The standard contrast agent used in the majority of studies was a bolus of 0.075 mmol/kg gadobutrol (Gadovist, Bayer AG, Leverkusen, Germany) administered at 4 ml/s with 20 ml saline flush during image acquisition. Scanners from Siemens Healthineers or Philips scanners, with both 3T and 1.5T field strengths, were utilised.

Image extraction occurred in two stages and underwent review by a level 3 CMR reader. Stage 1 involved selecting the peak LV cavity signal intensity frame using an automated pipeline based on sum and peak pixels per frame. Stage 2 included cropping the image to encompass only the LV myocardium and cavity using centre crop function. Unique IDs for each case were used to link image series with corresponding clinical data.

### 3.4 Neural networks building

#### 3.4.1 Defining labels

Positive ischaemia was identified in the CMR reports using the AHA 16-segment model, categorising each segment as either positive or negative without ordinal or transmuralilty quantification. Binary labels were employed to annotate images for training AHA plot classifiers. Similarly, binary values were used for all-cause mortality in training the outcome prediction model.

#### 3.4.2 AHA plot classifier

**Fig. 1:**
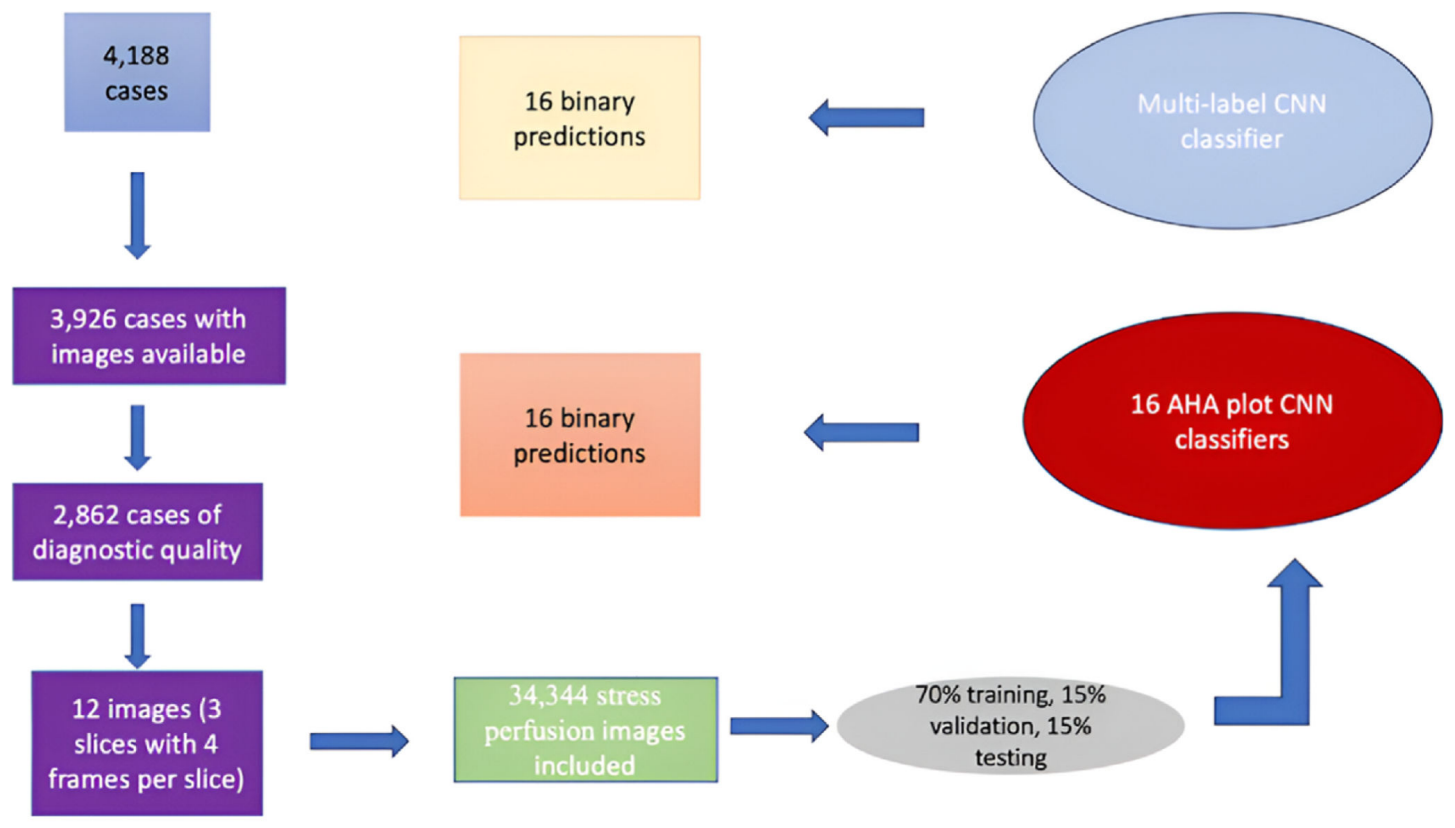
A diagram showing data extraction process for AHA classifiers. AHA; American Heart Association, CNN; convolutional neural networks.

A CNN architecture was employed for training AHA plot classifiers. Different experiments were conducted with different CNN architectures, utilising two approaches: a multi-label classifier training one CNN for all 16 labels, and a cluster of binary classifiers with individual CNNs for each AHA segment. The choice of data extraction and neural network configurations is depicted in Fig. 1.

To determine the optimal architecture, an experiment was conducted on AHA segment 1 (basal anterior) using five networks: LeNet [13], AlexNet [14], VGG19 [15], ResNet50 [16] and GoogleNet [17]. The best-performing design was then used for the remaining binary classifiers and the multi-label classifier. Training progress was monitored with early stopping based on the validation precision/recall curve or F1 score. Two optimisers, Adam and Stochastic Gradient Descent (SGD), were tested with various learning rates. Data were split into 70 % for training, 15 % for validation and 15 % for testing.

All images were resized to a uniform height and width of 224 pixels, and frames were stacked for each case with an input shape of (224, 224, 12). As each AHA segment had binary classes (0 or 1), binary cross-entropy loss function was used, and the number.

of classes was set to 1. A batch size of 64 was employed with a maximum of 500 training epochs and early stopping after achieving the best F1 score in the validation set with a patience of 50 epochs. Adam optimiser with a learning rate of 0.001 was ultimately chosen.

#### 3.4.3 Hybrid neural network

Hybrid neural network (HNN) was developed to incorporate mixed input data from images and clinical data for predicting mortality outcomes. CNN architecture was used to extract features from stress perfusion images, removing the top prediction layer and flattening the output to a Dense shape of 4. A Multi-Layer Perceptron (MLP) with 2 Dense layers was employed to extract features from continuous and categorical clinical variables, also removing the top prediction layer and flattening the output to a Dense shape of 4, ensuring compatibility with the CNN output. Clinical variables used for feature extraction included age, gender, CKD, HTN, heart failure, smoking history, dyslipidaemia, DM, and CVA.

Both outputs were concatenated and passed to 2 Dense layers for binary mortality prediction in the final layer. Various CNN architectures were tested, following the AHA classifiers approach.

### 3.5 Statistical analysis

Categorical variables were presented as numbers and percentages, while continuous variables were expressed as means and standard deviations. Follow-up duration was calculated as the mean time to the all-cause mortality events, with cases without events and those with a shorter duration from the CMR date to the collection date being excluded. The population was stratified into three age subgroups (<65 years, 65–75 years, >75 years) due to variations in cardiovascular disease risks among different adult age categories [18] Baseline characteristics, clinical risk factors and CMR data differences among subgroups were assessed using the Chi-Square test for categorical variables and One Way ANOVA for continuous variables. P value of <0.05 was considered statistically significant.

For binary classifiers, class weight was used during training and calculated as:

1/n∗N/2

where n is number of examples per class, and N is the sample size. Class weights were incorporated during model training to address class imbalance.

Performance metrics for models included accuracy, recall, precision, area under the curve (AUC); and F1 score. The total AUC for AHA classifiers was calculated as the micro-average value. Models’ performance was compared using McNemar’s test, and agreement between results and ground truth reports findings was assessed using the Cohen Kappa score for binary classification.

All analysis were conducted using the Python programming language, version 3.10, with the Tensorflow library employed for model building and training.

## 4 Results

### 4.1 Baseline characteristics

Table (1) provides an overview of all baseline characteristics, CMR data, and clinical risk factors.

The study included a total of 2862 cases, with 223 patients (8 %) experiencing positive mortality events. The mean follow-up period was 1090 days. Males constituted 65 % of the study population, and 3 T field strength CMR scanners were more commonly used (62 % vs 38 % for 1.5 T). Adenosine was the predominant vasodilator agent used in stress studies (89 % vs 11 % for Regadenoson).

**Fig. 2:**
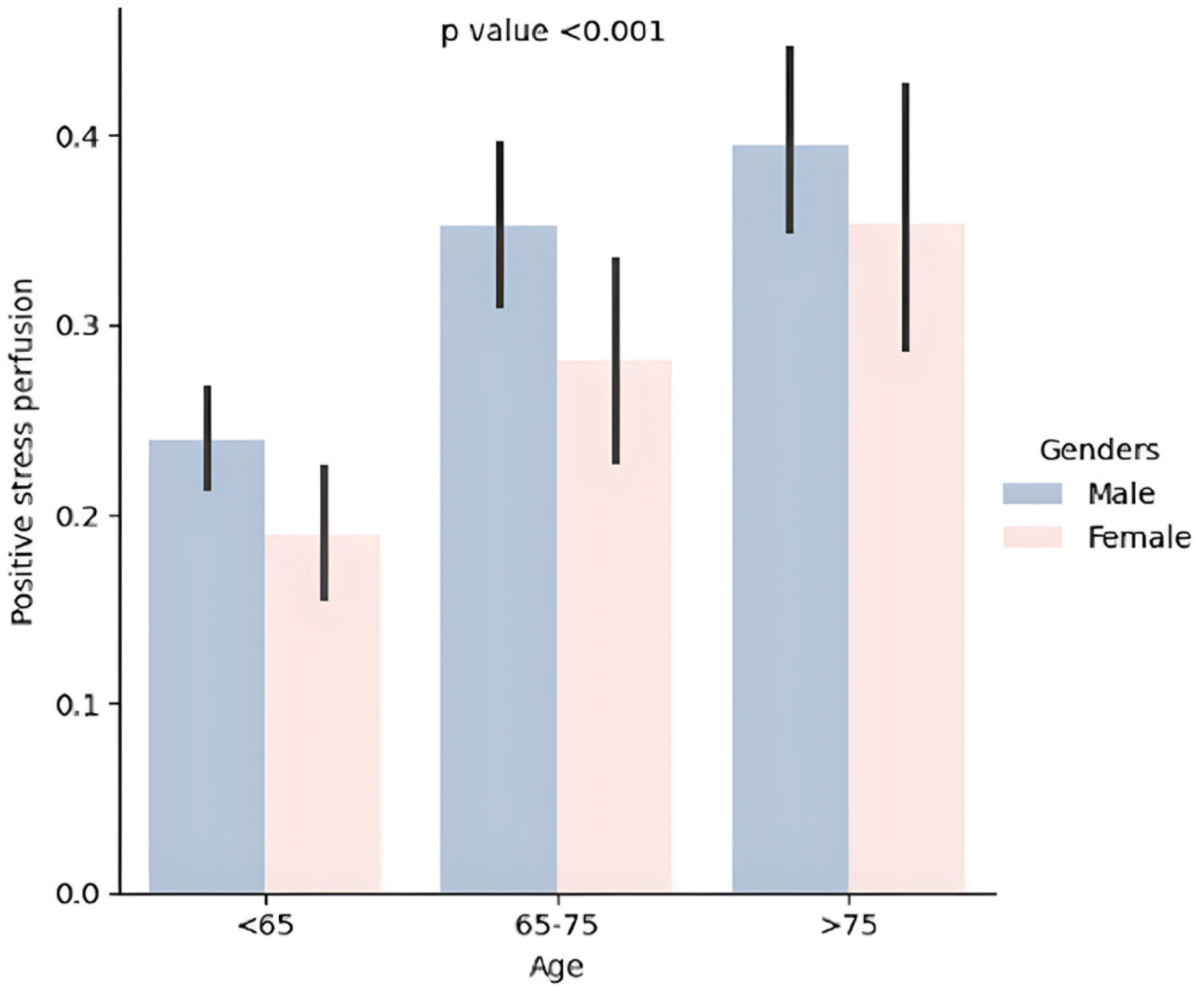
Barplot showing different age groups with gender categories and comparison based on positive stress perfusion.

Of the total cases, 810 (28 %) had at least one AHA segment positive for ischaemia, with 96 (12 %) experiencing mortality events. When stratified into three age subgroups, the older population (>75 years) exhibited a higher percentage of positive stress perfusion (see Fig. 2).

**Fig. 3:**
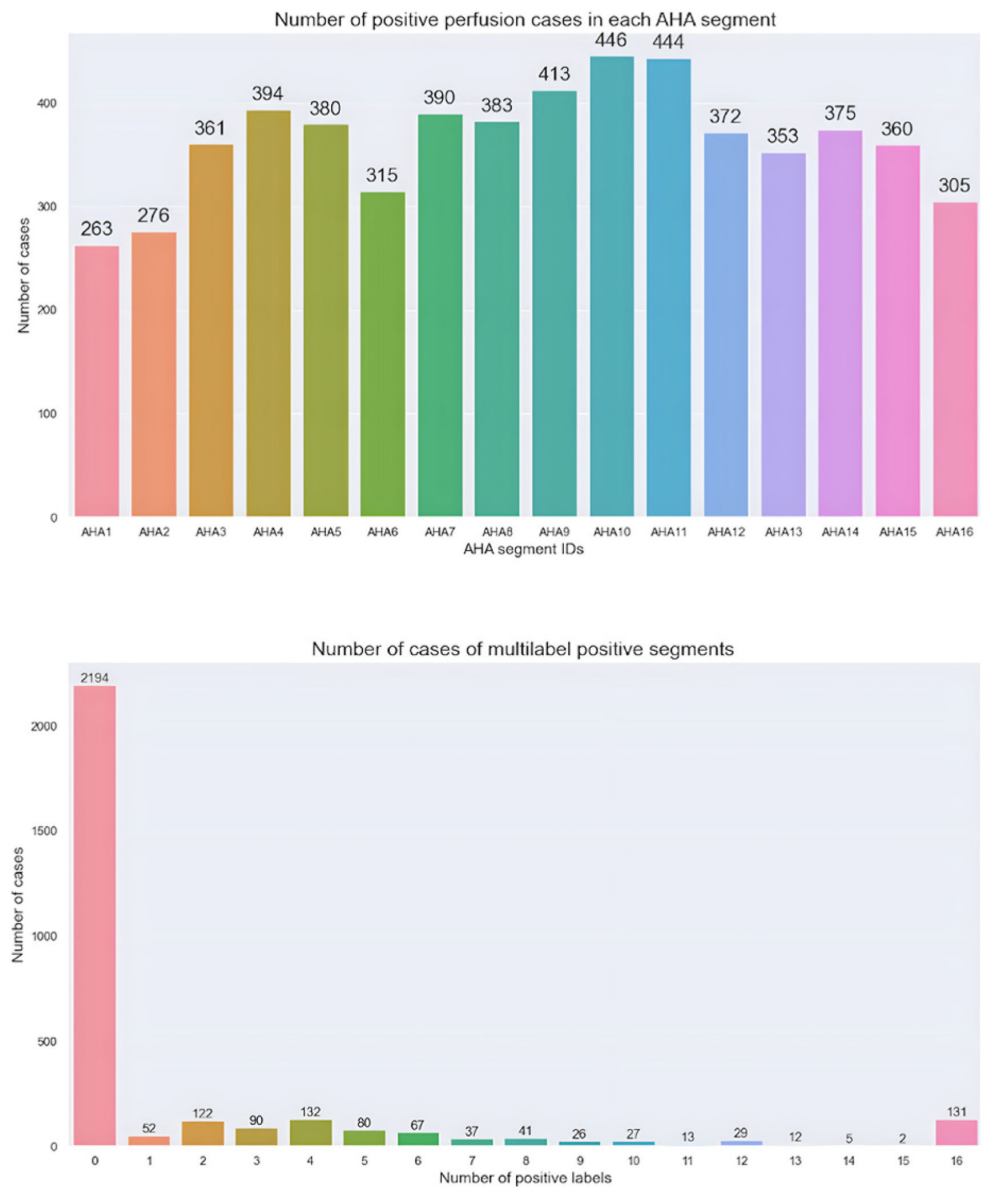
Categorical barplots showing different AHA segments with the number of positive cases in each category *(top)* and the number of cases with combined positive segments *(bottom)*.

Variation in positive perfusion cases were observed across individual AHA segments, with segment 10 (mid inferior) being the most commonly positive (446 cases) and segment 1 (basal anterior) being the least commonly positive (263 cases). Combining all segments, normal studies (all AHA segments with a score of 0) were the most prevalent (2194 cases, 76.67 %), while studies with 15/16 positive segments were the least common (only 2 cases). Further details are presented in Fig. 3.

### 4.2 Neural networks training

**Fig. 4:**
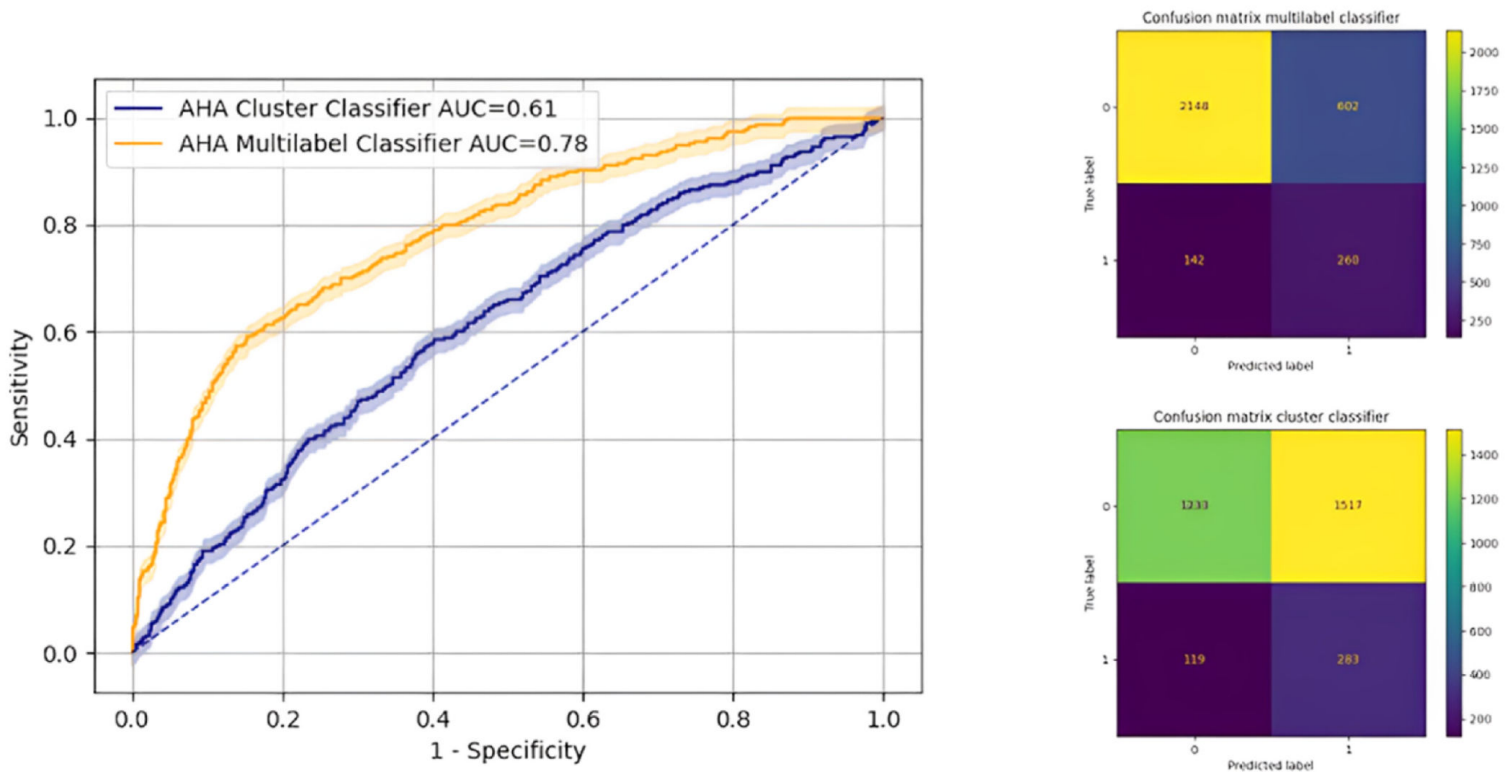
AUC with 95 % confidence interval comparing between cluster classifier and multilabel classifier (*left*). Confusion matrices are shown (*right*): multilabel classifier (*top*) and cluster classifier (*bottom*). AHA; American Heart Association, AUC; area under the curve.

The ResNet50 neural network emerged as the best-performing for AHA plot classification. Due to significant class imbalance with normal segments, the cluster classifier showed an AUC of 61 %, while the multi-label classifier exhibited superior performance with a micro-average AUC of 78 % (McNemar test P value < 0.001), as illustrated in Fig. 4.

**Fig. 5:**
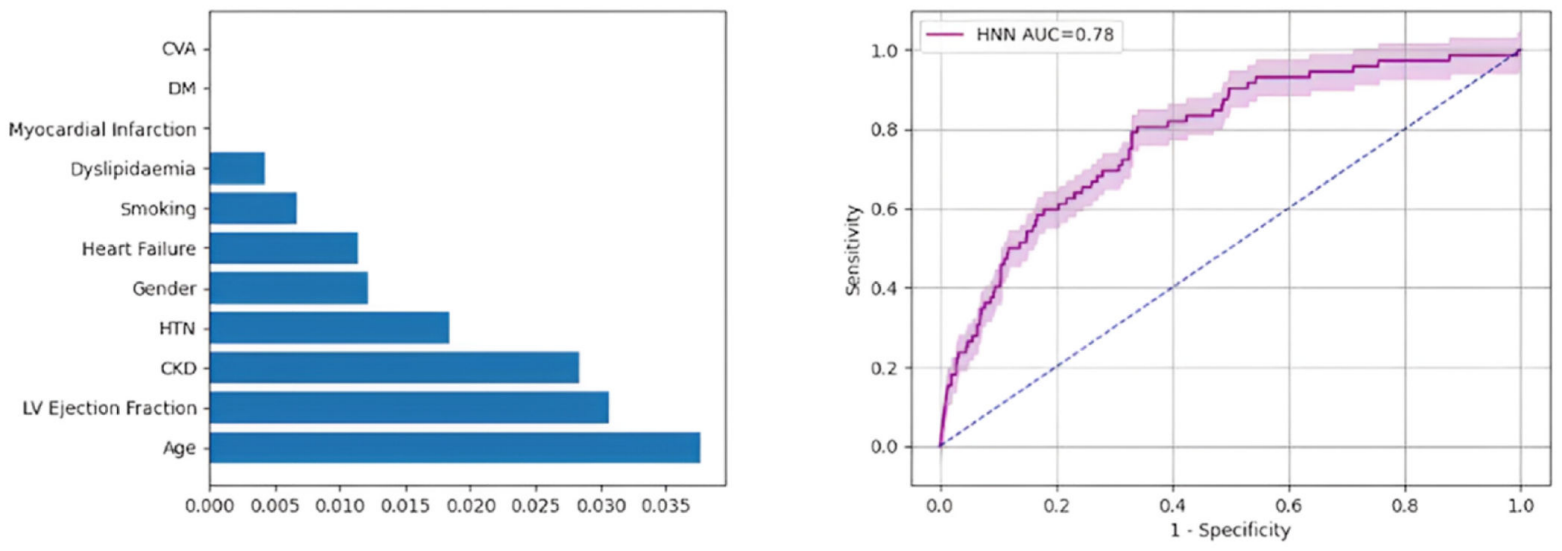
Information gain for mortality predictors using multivariate regression (*left*), and AUC with 95 % confidence interval for mortality HNN (*right*). AUC; area under the curve, CKD; chronic kidney disease, CVA; cerebrovascular accident, DM; diabetes mellitus, HNN; hybrid neural network, HTN; hypertension.

For image feature extraction in HNN, GoogleNet proved to be the most effective, achieving an AUC of 78 %. Logistic regression highlighted predictors information in descending order of information gain: age, LV ejection fraction, CKD, HTN, gender, heart failure, smoking and dyslipidaemia (Fig. 5). Table (2) provides a comprehensive over-view of performance metrics for all neural networks.

Comparing the classifiers with level 3 CMR readers from the reported ground truth binary values, the Cohen Kappa score indicated fair agreement (0.29) for multilabel classifier and no agreement for cluster classifier (0.06). An example of a prediction case involving RCA ischaemia with a mortality event is depicted in graphical abstract, where 5/16 positive ischaemic segments were correctly predicted.

## 5 Discussion

Image classification plays a crucial role in interpreting stress perfusion images, providing clinicians with valuable insights into myocardial function and identifying patients at risk of cardiac disease. Advances in machine learning and deep learning continue to drive improvements in accuracy and efficiency in this field, and medical literature has shown promising applications and techniques in various imaging modalities [9].

Stress perfusion CMR offers a rapid and robust assessment of myocardial ischaemia, proving highly accurate for CAD detection. However, its visual interpretation is complex and time-consuming, dependent on factors such as operator experience, stress perfusion sequence type, vendor, and image quality. These challenges limit widespread adoption, particularly in less-experienced centres [5]. This study demonstrates the feasibility of AI-based image classification for stress perfusion images using AHA segmentation, achieving an average AUC of 78 %.

The reported AUC of 78 % suggests that AI-based classification of stress perfusion CMR images is promising. The use of challenging legacy image data, often replaced by high-resolution sequences in modern practice, underscores the robustness of the model. While the Cohen-Kappa score indicates only fair agreement with human readers, the heavy class imbalance towards normal AHA segments (76.67 %) poses a significant challenge. Despite this, the CNN classifiers successfully learned features and classified images, hinting at the potential with improved data quality.

Advancements in imaging technology, including higher resolution, improved acceleration techniques, and enhanced computational power, have made non-invasive imaging more accessible. High-resolution modern stress perfusion sequences improve workflow efficiency and diagnostic accuracy. Training AHA classifiers on high quality images could enhance automated reporting functions. Balancing the dataset with more positive cases reporting ischaemia could further improve results. As performance improves, automated AI pipelines may replace repetitive tasks in the diagnostic process.

Prediction of mortality in CAD is crucial for treatment decisions. This study demonstrates the feasibility of incorporating image pixel data for outcome prediction using deep learning with feature extraction. Integrating image data with clinical data achieved a good performance level with an AUC of 78 %. This suggests the potential for mixed data types to enhance predictive capabilities in clinical practice.

## 6 Conclusion

This study demonstrates the feasibility of AI-based image classification using AHA segmentation, even with legacy images and imbalanced datasets. The potential for improved performance with modern sequences suggests that AI could automate and enhance the efficiency of CMR image reporting in daily clinical workflows.

The introduction of mortality prediction from mixed data types using hybrid neural networks is a novel and promising approach. The combination of qualitative AHA plot and mortality prediction offers a fully automated classification and prediction pipeline with potential clinical applications.

## 7 Practical and social implications

By leveraging the potential of AI-assisted stress perfusion CMR image classification, this can provide valuable tools for diagnosing and managing patients and lead to more timely interventions, improved patient outcomes, and potentially reduced healthcare costs. It can reduce the burden on radiologists and cardiologists, allowing for improved work-flow efficiency and reduce waiting times for diagnostic tests reporting.

Automated analysis of stress perfusion can facilitate remote diagnosis and consultation, and expand access to timely and high-quality cardiovascular care for patients in remote or rural communities.

As with any technology in healthcare, there are ethical considerations associated with stress perfusion image classification, including patient privacy, data security, and algorithm transparency. This requires thoughtful consideration of these ethical issues, as well as ongoing collaboration between healthcare stakeholders, policymakers, and technology developers to address potential biases and mitigate unintended consequences.

## 8 Limitations and future research

The primary limitation lies in the utilisation of legacy stress perfusion images with limited quality, impacting model performance during testing. The presence of non-diagnostic images led to a considerable exclusion of cases from baseline sample. The replacement of most legacy sequences with high-resolution ones in current practice may limit the applicability of the models to modern datasets.

The heavy class imbalance, with the majority of cases having normal segments, poses challenges for the model in predicting positive abnormal segments in external validation datasets. This imbalance may influence the model’s generalisability and sensitivity to abnormal findings. Future research would benefit from more balanced and high quality datasets.

The dataset predominantly includes images acquired using 3T field strength from Siemens and Philips scanners. Generalising of the model to different field strengths or vendors should be approached with caution, as variations in acquisition parameters and hardware could impact the model’s performance.

The variable follow-up periods introduce complexity to the analysis, potentially affecting predictive binary models. Future research should consider using data from randomised controlled trials (RCTs) to enhance the reliability of outcome predictions.

## Figures and Tables

**Table 1:** Baseline characteristics by age subgroups. Values are presented as number (%) for categorical variables, mean ± standard deviation for continuous variables. AF = atrial fibrillation; CKD = chronic kidney disease; CVA = cerebrovascular accident; DM = diabetes mellitus; HB = heart block; HTN = hypertension; LGE = late gadolinium enhancement; LVEF = left ventricular ejection fraction; MI = myocardial infarction; RVEF = right ventricular ejection fraction; T = tesla; VF = ventricular fibrillation; VT = ventricular tachycardia.

	Total (n = 2862)	<65 years (n = 1414)	65–75 years (n = 853)	>75 years (n = 595)	P value
Death	223 (8)	32 (2)	66 (8)	125 (21)	<0.001*
Sex					
Male	1859 (65)	919 (65)	540 (63)	400 (67)	0.306
Female	1003 (35)	495 (35)	313 (37)	195 (33)	
Clinical risk factors					
Smoking	362 (13)	140 (10)	138 (16)	84 (14)	<0.001*
DM	125 (4)	54 (4)	40 (5)	31 (5)	0.326
HTN	1108 (39)	462 (33)	390 (46)	256 (43)	<0.001*
Dyslipidaemia	590 (21)	245 (17)	224 (26)	121 (20)	<0.001*
CVA	239 (8)	92 (7)	90 (11)	57 (10)	0.002*
CKD	147 (5)	36 (3)	55 (6)	56 (9)	<0.001*
Previous MI	725 (25)	346 (24)	232 (27)	147 (25)	0.325
Heart failure	476 (17)	183 (13)	169 (20)	124 (21)	<0.001*
Arrhythmia					
AF	427 (15)	136 (10)	149 (17)	142 (24)	<0.001*
Atrial flutter	112 (4)	42 (3)	46 (5)	24 (4)	0.016*
VT	209 (7)	85 (6)	66 (8)	58 (10)	0.011
VF	30 (1)	16 (1)	10 (1)	4 (1)	0.597
Field strength					
1.5T	1076 (38)	549 (39)	307 (36)	220 (37)	0.643
3T	1788 (62)	864 (61)	546 (64)	378 (64)	
Stress agent					
Adenosine	2554 (89)	1272 (90)	767 (90)	515 (86)	0.017*
Regadenoson	308 (11)	142 (10)	86 (10)	80 (14)	
LVEF	55 ± 13	57 ± 11	54 ± 14	51 ± 15	<0.001*
RVEF	58 ± 10	58 ± 09	59 ± 10	57 ± 11	0.008*
+ve ischaemia	810 (28)	302 (21)	281 (33)	227 (38)	<0.001*
+ve LGE	893 (31)	307 (22)	301 (35)	285 (48)	<0.001*

**Table 2:** Comparison of performance metrics for all CNN models. AUC; area under curve, CNN; convolutional neural network, CK; Cohen Kappa, HNN; hybrid neural network.

	Accuracy	Precision	Recall	AUC	F1 Score	CK score
Cluster Classifier	0.48	0.17	0.60	0.61	0.18	0.06
Multilabel Classifier	0.76	0.26	0.75	0.78	0.40	0.29
HNN	0.68	0.16	0.77	0.78	0.26	na
